# Phosphate uptake kinetics and tissue-specific transporter expression profiles in poplar (*Populus* × *canescens*) at different phosphorus availabilities

**DOI:** 10.1186/s12870-016-0892-3

**Published:** 2016-09-23

**Authors:** Mareike Kavka, Andrea Polle

**Affiliations:** 1 Forstbotanik und Baumphysiologie, Georg-August Universität Göttingen, Büsgenweg 2, 37077 Göttingen, Germany; 2 Labor für Radio-Isotope, Georg-August Universität Göttingen, Büsgenweg 2, 37077 Göttingen, Germany

**Keywords:** Phosphate transporter, Poplar, Uptake kinetics, Expression profile

## Abstract

**Background:**

Phosphorus (P) is a major plant nutrient. It is transported into and allocated inside plants by four families of phosphate transporters (PHT1 to PHT4) with high or low affinity to phosphate. Here, we studied whole-plant P uptake kinetics and expression profiles of members of the PHT families under high, intermediate and low P availability in the woody crop poplar (*Populus* × *canescens*) in relation to plant performance.

**Results:**

Poplars exhibited strong growth reduction and increased P use efficiency in response to lower P availabilities. The relative P uptake rate increased with intermediate and decreased with low P availability. This decrease was not energy-limited because glucose addition could not rescue the uptake. The maximum P uptake rate was more than 13-times higher in P-starved than in well-supplied poplars. The K_m_ for whole-root uptake ranged between 26 μM and 20 μM in poplars with intermediate and low P availability, respectively. In well-supplied plants, only low uptake rate was found. The minimum concentration for net P uptake from the nutrient solution was 1.1 μM. All PHT1 members studied showed significant up-regulation upon P starvation and were higher expressed in roots than leaves, with the exception of *PtPHT1;3. PtPHT1;1* and *PtPHT1;2* showed root- and P starvation-specific expression. Various members of the PHT2, PHT3 and PHT4 families showed higher expression in leaves than in roots, but were unresponsive to P deprivation. Other members (*PtPHT3;1*, *PtPHT3;2*, *PtPHT3;6*, *PtPHT4;6* to *PtPHT4;8*) exhibited higher expression in roots than in leaves and were in most cases up-regulated in response to P deficiency.

**Conclusions:**

Expression profiles of distinct members of the PHT families, especially those of PHT1 were linked with changes in P uptake and allocation at whole-plant level. The regulation was tissue-specific with lower P responsiveness in leaves than in roots. Uptake efficiency for P increased with decreasing P availability, but could not overcome a threshold of about 1 μM P in the nutrient solution. Because the P concentrations in soil solutions are generally in the lower micro-molar range, even below the apparent K_m_-values, our findings suggest that bare-rooted poplars are prone to suffer from P limitations in most environments.

**Electronic supplementary material:**

The online version of this article (doi:10.1186/s12870-016-0892-3) contains supplementary material, which is available to authorized users.

## Background

Phosphorus (P) is a major nutrient, required for growth and metabolism, but often is the least plant-available compound in soil [1]. Plants take up phosphorus in the form of inorganic phosphate (P_i_), whose concentration is usually low (<10 μM) in soil solutions [2]. P uptake and translocation is achieved by a diverse group of phosphate transporters (PHTs), which cluster in four families [3, 4]. Most members of family 1 (PHT1) are localized in the plasma membrane [5], of PHT2 in chloroplasts [6], of PHT3 in the mitochondria [4] and of PHT4 in various subcellular compartments, including heterotrophic plastids and the Golgi apparatus [3]. In *Arabidopsis*, members of the PHT1 family exhibit strong expression in roots, are responsible for P uptake from soil and for the distribution and remobilization within the plant [5, 7], whereas the members of the other PHT families are required for intracellular P distribution [3, 6, 8].

Kinetic measurements showed that plants possess two different P uptake systems: high affinity systems that respond to the P status of the plant, and low affinity systems that are expressed constitutively [9]. Low affinity P uptake systems are operational under high P availabilities and exhibit K_m_ values of 50 to several hundred μM [9]. In *Arabidopsis*, PHT2;1 [6, 10] and all transporters of family 4 [3] were shown to be low affinity transporters. But also family 1 may contain low affinity transporters (rice: OsPHT1;2 [11], barley: HvPHT1;6 [12]). The expression of high affinity transporters is induced when P availability is low [13]. Their K_m_ values are in the lower micro-molar range [14]. For example, *Arabidopsis* AtPHT1;1 exhibited a K_m_ of 3 μM [15] and transported together with AtPHT1;4 the highest proportion of phosphate into the roots under low P conditions [16].

At the whole-plant level, P uptake is driven by the interplay of multiple P transporters and internal resource allocation. Whole-plant P uptake has mainly been determined for agricultural crops such as barley, maize, potato and soybean, and was about 1.8 to 5.2 times higher in P starved than in P sufficient plants [17–20], but information on the involved transport systems is lacking.

*Populus* species are important woody crops [21, 22]. With an increasing demand for woody biomass, an extension of poplar plantations is expected, especially on marginal soils where nutrients are often limited [21, 22]. Previous studies on selected members of the PHT1 family in poplar demonstrated up-regulation when the trees were exposed to low or no P in the growth medium [23, 24]. It was further shown that the K_m_ for P accumulation in roots of P starved poplars (*P. tremuloides*) was 32 μM [25]. P uptake kinetics that link changes in P uptake with changes in the expression profiles in different PHT families, are still missing. Furthermore, it is not known whether modulation in P availability affects P acquisition, internal allocation and P use efficiency in poplar.

The goal of the present study was to characterize whole-plant P uptake kinetics and expression profiles of the PHT transporter families 1, 2, 3, and 4 in roots and leaves under high, intermediate and low P availability in poplar (*P*. × *canescens*). As pre-requisite for these studies we annotated the poplar PHT families 2, 3, and 4 in addition to the known PHT1 family [24]. We further determined P acquisition and allocation in response to different P availabilities. Our results show that intermediate P concentrations of about 6 μM, which are often present in environments, led to a tissue-specific regulation of PHTs, a relative increase in P uptake with low K_m_ and high v_max_, increased P use efficiency, but strong growth reduction.

## Methods

### Plant material and growth conditions

In vitro micropropagated [26] *Populus* × *canescens* (INRA717 1-B4) plantlets were grown on half-strength Murashige and Skoog medium for three weeks to develop roots. Afterwards, they were planted singly into PVC tubes (5 cm diameter; 40 cm length) with one drain [27, 28] filled with autoclaved sand (Ø 0.4–3.15 mm particle size, Melo, Göttingen, Germany) and grown in a greenhouse at an air humidity of about 65 %. The plants were supplemented with additional light (EYE Clean Ace MT400DL/BH, EYE Lighting Europe, Uxbridge, UK; 50–100 μmol quanta m^−2^ s^−1^of photosynthetically active radiation, depending on poplar height and natural light condition) for 14 h a day from 7 am to 9 pm. The plants were automatically irrigated as described by Müller et al. [28] every 4 hours (ca. 6.5 mL, after 45 days ca. 9 mL) with Long Ashton nutrient solution [29] containing either high phosphate (HP) supply (200 μM KNO_3_, 900 μM Ca(NO_3_)_2_, 300.2 μM MgSO_4_, 599.9 μM KH_2_PO_4_, 41.3 μM K_2_HPO_4_, 10 μM H_3_BO_3_, 2 μM MnSO_4_, 7 μM Na_2_MoO_4_, 40 nM CoSO_4_, 200 nM ZnSO_4_, 128 nM CuSO_4_, 10 μM EDTA-Fe, in total: 641 μM P_i_) or reduced P_i_ concentrations. Mildly P_i_ starved (medium phosphate, MP) poplars received Long Ashton solution with 5.999 μM KH_2_PO_4_ and 0.413 μM K_2_HPO_4_ and additionally 675.8 μM KCl and P_i_ starved (low phosphate, LP) plants 0.060 μM KH_2_PO_4_ and 0.004 μM K_2_HPO_4_ and additionally 682.5 μM KCl. Plant height was measured weekly from the stem base to the apex. This experiment was repeated four times for different measurements with 12 biological replicates each time.

### Labeling of the poplars with ^33^P and harvest

Sixty-day-old HP, MP and LP poplars (*n* = 5 per treatment, experiment 2) were watered by hand at 7 am and 10 am with the respective nutrient solution (16 and 14 mL for HP, 4 mL for MP and 2 mL for LP) and labeled with H_3_^33^PO_4_ (Hartmann Analytic, Braunschweig, Germany) at 11 am: 20.25 μL of the H_3_^33^PO_4_ stock solution were mixed with 5.5 mL of each of the respective nutrient solutions and 1 mL of each labeling solution was applied once to the poplars. This treatment resulted in 1.2 (±0.013 resp. 0.002) MBq for the HP and MP plants and 1.12 (±0.034) MBq for the LP plants (mean ± SE, *n* = 3). The specific radioactivity was 1.88 × 10^3^ Bq nmol^−1^ P for HP, 1.87 × 10^5^ Bq nmol^−1^ P for MP and 1.46 × 10^7^ Bq nmol^−1^ P for LP poplars.

The automatic irrigation was stopped during the chase period of two days. During this time the poplars were irrigated by hand with unlabeled nutrient solution avoiding through-flow. Two days after label application, the plants were harvested. The roots were briefly washed with tap water. Each plant was divided into fine roots, coarse roots, stem and leaves. The biomass of the tissues was determined immediately after harvest and after drying at 60 °C for 7 days.

### Phosphorus distribution at the whole-plant level

To visualize the distribution of radioactivity, the poplars were dried at 60 °C for one day pressed between paper and two glass plates. Autoradiographs were taken with a Phosphorimager (FLA 5100, Fuji, Japan) after exposure for 30 min on imaging plates (Imaging Plate BAS-MS 2040, 20 × 40 cm, Fuji, Japan). The image was taken with the program Image Reader FLA-5000 (version 3.0, Fuji Film, Japan) with 100 μm resolution and analyzed with AIDA Image Analyzer (version 4.27, raytest Isotopenmeßgeräte, Straubenhardt, Germany).

### Determination of net ^33^P and total P uptake

Dried plant tissues were milled (Retsch, type MM2, Haan, Germany) to a fine powder. About 25 mg of leaf or stem powder and 10 mg of fine or coarse root powder were weighed into glass vials (20 mL, PerkinElmer Life and Analytical Sciences, Rodgau, Germany) and incinerated for 4 h at 500 °C (M104, Heraeus Holding, Hanau, Germany). The residual material was mixed with 10 mL of scintillation liquid (Rotiszint® eco plus [Carl Roth, Karlsruhe, Germany]) and the radioactivity was measured by liquid-scintillation technique (Tri-Carb® 2800TR [PerkinElmer Life and Analytical Sciences, Rodgau, Germany]). The maximum count time was 10 min in normal count mode calculated against a quench set of ^33^P. All values were corrected for the half-life of ^33^P. ^33^P-activity in plants and proportion of ^33^P-uptake were calculated using following equations:

$$ \begin{array}{l}\mathrm{Radioactivity}\ \mathrm{in}\ \mathrm{plant}\ \mathrm{tissue}\ \left[\mathrm{Bq}\right]=\\ {}\left(\mathrm{activity}\ \mathrm{in}\ \mathrm{vial}\ \left[\mathrm{Bq}\right]\hbox{-} \mathrm{background}\ \left[\mathrm{Bq}\right]\right)\times 0{.5}^{\frac{\mathrm{date}\ \mathrm{of}\ \mathrm{harvest}\hbox{-} \mathrm{date}\ \mathrm{of}\ \mathrm{measurement}}{25.3}}\times \frac{\mathrm{dry}\ \mathrm{mass}\ \mathrm{tissue}\ \left[\mathrm{g}\right]}{\mathrm{sample}\ \mathrm{in}\ \mathrm{vial}\ \left[\mathrm{g}\right]}\end{array} $$

$$ {}^{33}\mathrm{P}\hbox{-} \mathrm{recovery}\ \left[\%\right]=\frac{\mathrm{radioactivity}\ \mathrm{in}\ \mathrm{plant}\ \left[\mathrm{Bq}\right]}{\mathrm{given}\ \mathrm{radioactivity}\ \left[\mathrm{Bq}\right]}\times 100 $$

To determine total P uptake the specific radioactivity of the nutrient solution was used:

$$ \mathrm{P}\ \mathrm{uptake}\;\left[\mathrm{nmol}\right]=\frac{\mathrm{measured}\ \mathrm{radioactivity}\ \mathrm{in}\ \mathrm{plant}\ \left[\mathrm{Bq}\right]}{\mathrm{specific}\;\mathrm{radioactivity}\;\left[\mathrm{Bq}\;\mathrm{n}\mathrm{m}\mathrm{o}{\mathrm{l}}^{\hbox{-} 1}\mathrm{P}\right]}. $$

### Determination of total P contents

Dry fine root, coarse root, leaf and stem of 63-day-old HP, MP and LP poplars (*n* = 4 per treatment, experiment 1) were powdered and pressure-extracted in HNO_3_ [30]. Total phosphorus concentration was measured using an inductively coupled plasma optical emission spectrometer (ICP-OES; Optima 5300 DV, PerkinElmer Life and Analytical Sciences, Rodgau, Germany). P use efficiency (PUE) was calculated as dry mass per total P content. The equation for the whole plant PUE was

$$ \mathrm{P}\mathrm{U}\mathrm{E}\ \left[{\mathrm{g}\ \mathrm{mg}}^{\hbox{-} 1}\right] = \frac{{\mathrm{DM}}_{\mathrm{total}}}{\mathrm{P}\ {\mathrm{conc}}_{\mathrm{L}}\times {\mathrm{DM}}_{\mathrm{L}}+\mathrm{P}\ {\mathrm{conc}}_{\mathrm{S}}\times {\mathrm{DM}}_{\mathrm{S}}+\mathrm{P}\ {\mathrm{conc}}_{\mathrm{FR}}\times {\mathrm{DM}}_{\mathrm{FR}}+\mathrm{P}\ {\mathrm{conc}}_{\mathrm{CR}}\times {\mathrm{DM}}_{\mathrm{CR}}} $$

with DM: dry mass [g], P conc: total P concentration in fraction [mg g^−1^], L: leaves, S: stem, FR: fine roots, CR: coarse roots.

### Phosphate uptake after glucose supply and determination of carbohydrates

Seventy-seven-day-old HP, MP and LP poplars (*n* = 5 per treatment, experiment 3) were irrigated with 80 mL HP, MP or LP Long Ashton solution with or without 400 mM glucose at 5 am. Along with turning on the additional light at 7 am, they were labeled with H_3_^33^PO_4_ (Hartmann Analytic, Braunschweig, Germany) in 1 mL of HP, MP or LP Long Ashton solution to yielding 1.08 (±0.006) MBq in the growth medium of HP poplars and 1.09 MBq (±0.0008 resp. 0.003) in the growth medium of MP and LP poplars (mean ± SE, *n* = 2). At the end of the light period (9 pm), the poplars were harvested to determine biomass, ^33^P activity (as above), and the carbohydrate concentrations. Leaves (half of each leaf) and fine roots for carbohydrate determination were immediately shock frozen in liquid nitrogen and stored at −80 °C. The tissues were freeze-dried at −80 °C for three days (BETA1, Martin Christ Gefriertrocknungsanlagen, Osterode am Harz, Germany) and milled (as above). About 25 mg tissue powder was extracted in 1.5 ml dimethyl sulfoxide/hydrochloric acid (80:20 (v:v)) at 60 °C for 30 min. After centrifugation, 200 μL of the supernatant were mixed with 1250 μL 0.2 M citrate buffer (pH 10.6). After an additional centrifugation, 400 μL of the supernatant were mixed with 400 μL citrate buffer (50 μM, pH 4.6). Two hundred μL were mixed with 250 μL reaction solution (4 mM NADP, 10 mM ATP, 9 mM MgSO_4_, 0.75 M triethanolamine, pH 7.6) and 300 μL H_2_O. The reaction was started by subsequent addition of enzymes and the production of NADPH was determined photometrically at a wavelength of 340 nm and 25 °C until no further increase was observed. For glucose determination 10 μL (ca. 3.4 U; 1.7 U) of hexokinase; glucose-6-phosphate-dehydrogenase (Roche Diognostics, Mannheim, Germany), and subsequently for fructose 5 μL (ca. 17.5 U) of phosphoglucose-isomerase (Roche Diognostics, Mannheim, Germany) were added. To determine the amount of sucrose 400 μL (ca. 12 U) invertase (Sigma-Aldrich Chemie, Steinheim, Germany) solution (0.1 mg invertase in 1 mL 0.32 M citrate buffer, pH 4.6) were mixed with 400 μL of supernatant and then glucose was determined as above. Free glucose was subtracted. The concentration of glucose (fructose) was calculated for ΔE = E1-E0 with E1 and E0 being the extinction of assay after and before addition of glucose-6-phosphate-dehydrogenase (after and before addition of phosphoglucose-isomerase) and the extinction coefficient ε = 6.3 L mmol^−1^ cm^−1^ for NADPH. Sucrose concentration was below the detection limit in most of the samples. Each sample was measured in triplicate.

### Kinetic measurements

Roots of nine-week-old HP, MP and LP poplars (experiment 4) were washed carefully with tap water to remove the sand. The plants were transferred to aerated nutrient solution with HP, MP and LP Long Ashton nutrient solution and acclimated to this and lab conditions under continuous light (about 10 μmol quanta m^−2^ s^−1^ of photosynthetically active radiation, TLD18W/840, Philips Lighting, Hamburg, Germany) for 1 day. Phosphate uptake was determined with the method of Claassen and Barber [31]. The plants were removed from the nutrient solutions, the roots were cautiously surface-dried between tissue papers, washed with the experimental solution (see below), surface-dried again and placed in plastic beakers. An appropriate volume (5 to 45 ml) of Long Ashton nutrient solution which contained 213.7 μM P_i_ for all plants and ^33^P-phosphoric acid (Hartmann Analytic, Braunschweig, Germany) (to about 5375 Bq mL^−1^ of experimental solution) was added. During the time course of the experiment of up to 14 h, the nutrient solution was stirred and aerated with compressed air and water loss by plant transpiration was replaced by adding deionized water up to the marked original level. Uptake of P was calculated by the decrease in ^33^P in the experimental solution. For this purpose, 40 μL of experimental nutrient solution was removed at distinct time intervals and mixed with 0.5 mL inactive nutrient solution and 4 mL of Rotiszint® eco plus scintillation liquid (Carl Roth, Karlsruhe, Germany). Radioactivity was measured by liquid scintillation counting (Tri-Carb® 2800TR, PerkinElmer Life and Analytical Sciences, Rodgau, Germany). Samples were taken at min 0 (immediately after addition to the plants), 2.5, 5, 7.5, 10, 15, 20, 30, 45, 60 and 75 after addition to the plants, then every 30 min until 5 h, thereafter every hour and after 8 h every 2 h, if needed. The experiment lasted until the LP plants showed no further uptake, but at least 7 h. The experiment was conducted with 6 plants per P nutrient level. The plants were harvested at the end of the experiment and the biomass of the root system was measured.

The uptake rate (I_k,k+1_) was calculated between two time points (k and k + 1) with the following equations. Because the measured radioactivity fluctuated strongly in samples of the first minutes of the experiment, only the values after stabilization were used for calculations and models.

$$ {\mathrm{C}}_{\mathrm{k}}={\mathrm{C}}_0\times \frac{{\mathrm{A}}_{\mathrm{k}}}{{\mathrm{A}}_0} $$

C_k_: P concentration in the nutrient solution at time point k [μM]

A_k_: activity in nutrient solution at time point k [Bq μL^−1^]

C_0_: P concentration at time point 0 (start of experiment) [μM]

A_0_: activity in nutrient solution at time point 0 (start of experiment) [Bq μL^−1^]

$$ {\mathrm{I}}_{\mathrm{k},\mathrm{k}+1}=\frac{{\mathrm{C}}_{\mathrm{k}+1}\times \mathrm{V}-{\mathrm{C}}_{\mathrm{k}}\times \mathrm{V}}{\frac{{\mathrm{t}}_{\mathrm{k}+1}-{\mathrm{t}}_{\mathrm{k}}}{\mathrm{FM}}} $$

I_k,k+1_: P uptake rate between time point k and k + 1 [μmol min^−1^ g^−1^]

V: volume of experimental solution [L]

t_k,_ t_k+1,_ : time point k and k + 1 [min]

FM: fresh mass of fine roots [g].

Due to plant uptake, the P concentration in the nutrient solution declined during the experiment. Therefore, the P concentration (C_k,k+1_) for each uptake rate (I_k,k+1_) was calculated as:

$$ {\mathrm{C}}_{\mathrm{k},\mathrm{k}+1}=\frac{{\mathrm{C}}_{\mathrm{k}}+{\mathrm{C}}_{\mathrm{k}+1}}{2} $$

To model the change in uptake rate in relation to the concentration in the nutrient solution, I_k,k+1_ and the corresponding C_k,k+1_ were plotted und a curve was fitted to determine v_max_ (the maximum uptake rate), C_min_ (the minimum concentration at which the plants can take up phosphate) and K_m_ (the Michaelis-Menten constant) using the following equation in a non-linear model (R-package “nlme”, [32]):

$$ {\mathrm{I}}_{\mathrm{k},\mathrm{k}+1}=\frac{{\mathrm{v}}_{\max}\times \left({\mathrm{C}}_{\mathrm{k},\mathrm{k}+1}-{\mathrm{C}}_{\min}\right)}{{\mathrm{K}}_{\mathrm{m}}+{\mathrm{C}}_{\mathrm{k},\mathrm{k}+1}-{\mathrm{C}}_{\min }} $$

The fitted curve for these parameters was drawn; the standard errors of the predictions were used for calculation of the 95 %-prediction interval (R-package “emdbook” [33]). Because the uptake rate of the control plants (HP) was slow, a linear fit without slope was defined as v_max_. Outliers (>1.5 interquartile ranges below first and above third quartile) were excluded from the linear fit.

### RNA extraction and microarray analysis

The first three fully expanded leaves from the top and fine roots (<2 mm diameter) of 59-day-old HP, MP and LP poplars (experiment 2) were harvested and immediately shock frozen in liquid nitrogen. Six plants per treatment (i.e. 18 plants in total) were used and the leaves respective roots of two individual poplars were pooled yielding three biological replicates per treatment. Frozen tissue was milled in liquid nitrogen and RNA was extracted from about 1 g of plant powder according to Chang et al. [34] with modifications: no spermidine was used, 2 % β-mercaptoethanol was added separately from the extraction buffer and phenol:chloroform:isoamyl alcohol (Roti®-Aqua PCI, 25:24:1, Roth, Karlsruhe, Germany) was used instead of chloroform:isoamyl alcohol. RNA concentrations and purity were determined spectrophotometrically via the absorptions at 260 and 280 nm (BioPhotometer, Eppendorf, Hamburg, Germany). RNA integrity was determined with Agilent BioAnalyzer 2100 capillary electrophoresis at the Microarray Facility (MFT Services, Tübingen, Germany). Hybridization on the GeneChip® Poplar Genome Array (Affymetrix, Santa Clara, CA), washing, staining and scanning were conducted at the Microarray Facility (MFT Services, Tübingen, Germany). Raw and normalized data were uploaded into the EMBL-EBI ArrayExpress database [35] under E-MTAB-3934.

Statistical analyses of the raw data were performed as described in Janz et al. [36] using the free statistic software R (version 2.14.2 [37]). Only transcripts that had a detection call of “present” (“mas5calls” function with default settings for tau (0.015), alpha1 (0.04) and alpha2 (0.06)) on all replicate chips of at least one condition were used. For annotation of the microarray probe-set, the best gene model from the annotation file of the Aspen Data Base [38] was used. When several probe sets represented one gene, the mean value of the log_2_-expression data was used for further analysis. The log_2_-expression values x_i_ for each gene i of interest were normalized using z-transformation resulting in x_i_’:

$$ {{\mathrm{x}}_{\mathrm{i}}}^{,}=\frac{{\mathrm{x}}_{\mathrm{i}}-\overline{\mathrm{x}}}{\mathrm{s}}\mathrm{with} $$

$\overline{\mathrm{x}}$: arithmetic mean value of all log_2_-expression data for one gene,

s: standard deviation of all log_2_-expression data for one gene.

A heatmap of normalized expression values (“heatmap.2” function) was created using the R package “gplots” [39].

Here, the members of the putative PHT families 1, 2, 3, and 4 (according to [3, 4]) were retrieved as genes of interest. To obtain the gene IDs and protein sequences for all putative poplar PHT genes, BlastP searches in the database Phytozome v9.1 [40] were conducted. The genome of *Arabidopsis thaliana* [41], (https://phytozome.jgi.doe.gov/pz/portal.html#!info?alias=Org_Athaliana) was searched with the keyword “pht” extracting the amino acid sequences of eighteen annotated PHT genes. These sequences were used for a BlastP search (e-value cutoff at e-20) against the *Arabidopsis* genome. The Pfam database was used to identify proteins with functional domains [42]. Additional genes found by the BlastP search with the same domains (Mito_carr, MFS_1, Sugar_tr, Pfam-B_703, PHO4) that are present in the annotated proteins were added to the gene list. These protein sequences were used for BlastP searches against the *Populus trichocarpa* [43], the *Oryza sativa* [44], (https://phytozome.jgi.doe.gov/pz/portal.html#!info?alias=Org_Osativa) and the *Zea mays* genomes [45], (https://phytozome.jgi.doe.gov/pz/portal.html#!info?alias=Org_Zmays). When the resulting proteins had similar domains as *Arabidopsis* PHTs, they were used for a second BlastP against the respective genome. The resulting sequences were added to the list when the protein domains were similar to the PHT Pfam domains or when the best BlastP hit against the *Arabidopsis* genome was a PHT.

The phylogenetic tree was created with ClustalW2 and ClustalW2 phylogeny on the EMBL-EBI webpages [46], (http://www.ebi.ac.uk/Tools/msa/clustalw2/ and http://www.ebi.ac.uk/Tools/phylogeny/clustalw2_phylogeny) with default settings and displayed with MEGA6 [47], (http://www.megasoftware.net). Based on the phylogeny, the poplar PHT2 to PHT4 genes were named. Gene IDs of the members of all putative PHTs were compiled in Additional file 1: Table S1. The 1 kb upstream region of each poplar gene coding for a putative phosphate transporter was obtained from Phytozome using the BioMart tool on the Phytozome webpage (https://phytozome.jgi.doe.gov/biomart/martview) and used for a motif search with PLACE [48].

### Quantitative Real Time PCR of P transporter genes

RNA samples from the same samples that had been used for microarray analyses (200 ng μL^−1^ in 25 μL) were treated with Ambion® Turbo DNA-free™ kit (Life Technologies, Carlsbad, CA, USA) two times according to the manual instructions and transcribed to cDNA (0.5 μg) with the RevertAid First Strand cDNA Synthesis Kit and First Strand cDNA Synthesis Kit (Thermo Fisher Scientific, Braunschweig, Germany) using oligo(dT)-primers. For each gene, at least two technical replicates and three biological replicates were analyzed by quantitative Real Time PCR (qRT PCR) with a LightCycler 480® (Roche Diagnostics, Mannheim, Germany). The reaction volume (20 μL) consisted of 10 μL SYBR Green I Master kit (Roche Diagnostics, Mannheim, Germany), 2 μL of the forward and reverse primers (10 μM, Additional file 1: Table S2 for detailed information), 1 μL nucleic free water and 5 μL cDNA-solution (1:10 dilution). After pre-incubation (95 °C, 5 min), 45 or 55 cycles of amplification followed: 95 °C for 10 s, 57 °C (55 °C for *PtPHT1;2*) for 10 s and 72 °C for 20 s. Melting curve (95 °C for 5 s, 65 °C for 1 min, then to 97 °C at a rate of 0.11 °C s^−1^) analyses implemented in the LightCycler 480® software were used to assess primer specificity.

To calculate primer efficiency, raw data were converted using LC480 conversion (version 2014.1; www.hartfaalcentrum.nl/index.php?main=files&sub=LC480Conversion) and loaded into LinRegPCR (version 2016.0; [49]). The mean efficiency for each primer pair was calculated over all samples per gene after baseline subtraction. Cq-values were calculated using the fluorescence threshold of 3.597. Relative expression values for each sample were calculated against two reference genes (Potri.001G309500 [Actin], Potri.001G047200 [PPR-repeat gene]):

$$ \mathrm{Relative}\kern3pt \mathrm{Expression}=\frac{\sqrt{{\mathrm{E}}_{\left(\mathrm{R}\mathrm{e}{\mathrm{f}}_1\right)}^{\mathrm{Cq}}\times {\mathrm{E}}_{\left(\mathrm{R}\mathrm{e}{\mathrm{f}}_2\right)}^{\mathrm{Cq}}}}{{\mathrm{E}}_{\left(\mathrm{G}\mathrm{O}\mathrm{I}\right)}^{\mathrm{Cq}}} $$

E: efficiency of primer for gene

Cq: quantification cycle value of sample for gene

(GOI): gene of interest

(Ref_i_): reference gene i [50].

### Statistical analyses

R (versions 2.14.2 and 3.0.2; [37]) was used for all statistical analyses. Mean value ± standard error were calculated. One- or Two-Way-ANOVA and Tukey’s HSD were performed on original or transformed data. Residuals were tested visually for normal distribution and homogeneity of variance. Data were transformed logarithmically (log_2_) or by square root, if needed. For statistical comparisons of single kinetic parameters, Welsh’s *t*-test was performed using the output data of the models. For percentage data on ^33^P recovery and P allocation, a general linear model with binomial distribution was fitted on underlying count data, and an analysis of deviance was used to calculate significant factor and interaction effects. A subsequent Tukey test was performed to determine the homogenous subsets. Means were considered to differ significantly between treatments, if *p* ≤ 0.05. Differences between treatments are shown in figures and tables with different letters. The *p*-values calculated for gene expression data and v_max_ for kinetic data were adjusted by Bonferroni correction. Two-Way-Repeated measurement-ANOVA with Tukey’s HSD was performed for plant growth over time.

## Results

### Plant performance, P uptake and allocation in response to P deficiency

**Fig. 1:**
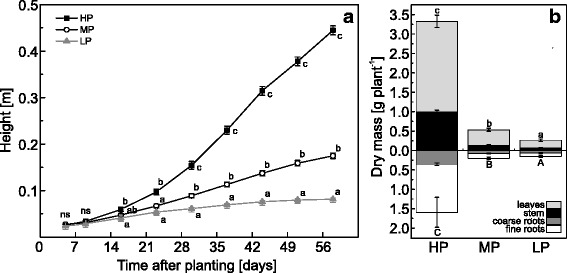
Growth characteristics of P deficient poplar. **a** Height of poplar plants grown with high (HP: 641 μM), medium (MP: 6.4 μM) or low (LP: 0.064 μM) phosphate concentrations in the nutrient solution. Different letters indicate significant differences between treatments at each measuring day (*p* ≤ 0.05, 2-Way-repeated measurement-ANOVA and Tukey’s honest significance test, *n* = 11-12, mean ± SE, ns: not significant). **b** Dry mass of poplar tissues after 60 days of growth with HP, MP or LP phosphate in the nutrient solution. Different letters indicate significant differences between treatments in above- and belowground tissues (*p* ≤ 0.05, ANOVA and Tukey’s honest significance test, *n* = 5, mean ± SE)

Pre-tests with five concentrations from 0.064 to 641 μM PO_4_^3−^ (further on called P) in the nutrient solution revealed a growth gradient for poplars with a strong decline in plant height and biomass between P concentration of 64 and 6.4 μM P (Additional file 1: Figure S1). We selected 641 (HP), 6.4 (MP) and 0.064 (LP) μM P in the nutrient solution for further studies because these concentrations resulted in high, intermediate and almost no growth during long-term cultivation (Fig. 1a) and because the highest P concentration is the typical P supply in nutrient solution [28], intermediate P is in the same range typically present in soil solutions and the lowest P concentration abolished growth entirely (Fig. 1a). In comparison with HP plants, biomass production of MP and LP poplars was significantly inhibited (Fig. 1b, Additional file 1: Figure S1).

**Fig. 2:**
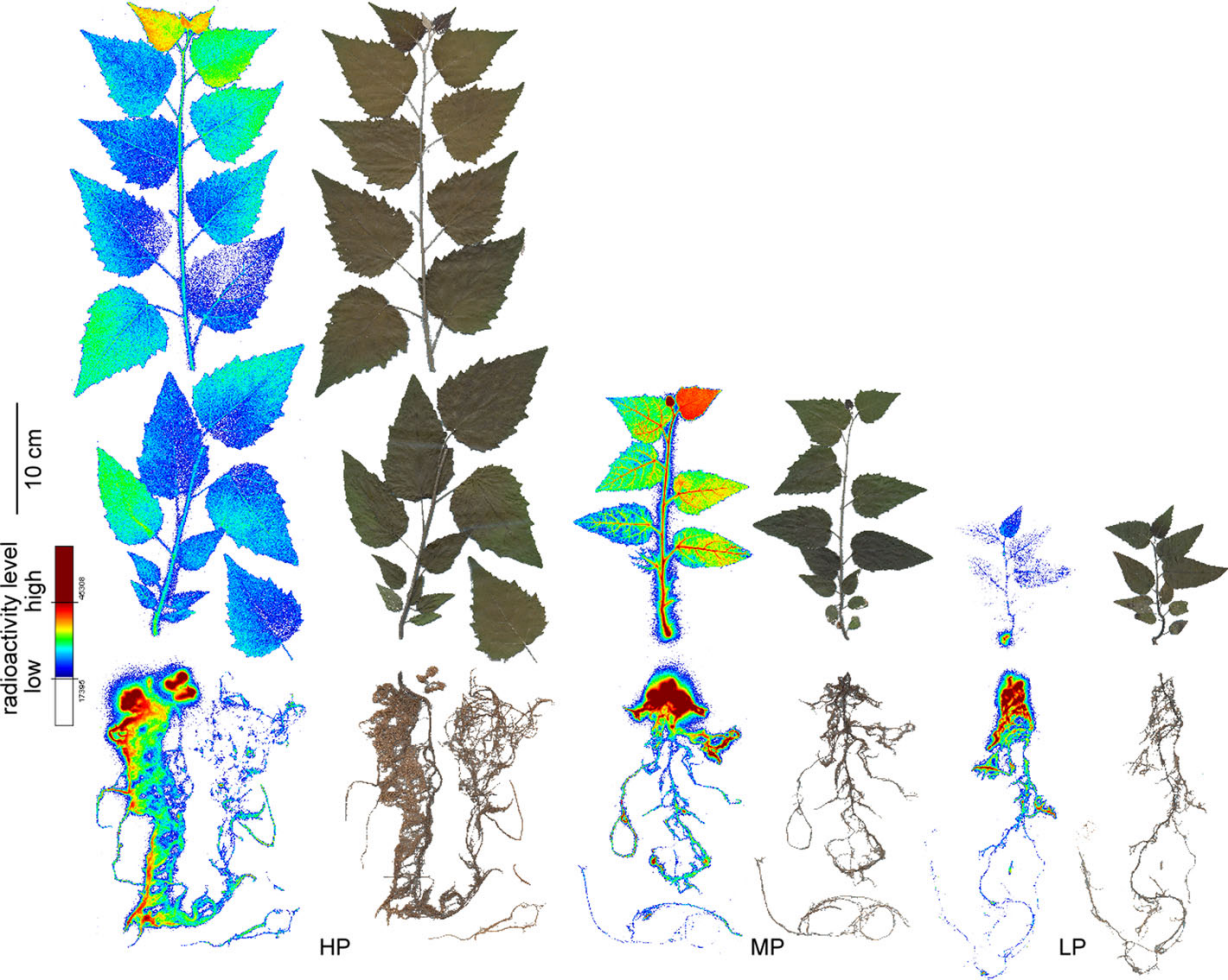
^33^P allocation in P deficient poplar. Autoradiographs of the ^33^P distribution in poplars grown with different P concentrations in the nutrient solution (HP: 641 μM, MP: 6.4 μM, LP: 0.064 μM) and scans of the corresponding plants. Autoradiographs were taken of whole plants 2 days after exposure to ^33^P as described under materials and methods. Uptake intensity is indicated by false color images with red indicating high and blue low radioactivity

HP plants exhibited the highest P concentrations, MP plants intermediate and LP plants the lowest P concentrations in all tissues (Table 1). Because our nutrient regime also resulted in growth decline, the whole-plant P content was about 30 times higher in HP plants than in MP and 50 times higher in HP compared with LP plants (Table 1). The relative allocation of P in above- to belowground material was significantly affected by P starvation with a higher fraction of P belowground after MP (about 50 %) and LP (43 %) than after HP treatments (37 %, *p* ≤ 0.05). Exposure of HP, MP, and LP plants to ^33^P in the nutrient solution showed the highest relative uptake for the MP plants. Within two days, they acquired 25 % of the P in the labeling solution, while HP plants took up about 15 % and LP only 2.5 % of the new P (Table 1). It was notable that highest P recovery of MP and LP plants was found in roots, whereas that of HP plants was more evenly distributed between roots and leaves (Table 1). The differences in distribution of the newly taken up ^33^P were also qualitatively confirmed by whole plant imaging (Fig. 2). While HP plants showed an almost even distribution across all tissues, MP plants showed a very high ^33^P concentration in the uppermost leaf and nearly no newly taken up P in the oldest leaves (Fig. 2). LP showed very little to no ^33^P uptake in older leaves and the main aboveground allocation to the youngest leaf, while root uptake was high. All plants showed strong accumulation of ^33^P in the roots, especially in parts adjacent to the root-stem junction (Fig. 2). Because of the different P concentrations in the nutrient solutions the calculated total P uptake (taking into account different dilution factors for ^33^P as detailed under materials and methods) corresponded to 95.7 nmol in HP plants, 1.63 in MP and 1.94 × 10^−3^ nmol in LP plants (Table 1). The P use efficiency was highest in LP plants (1.8 ± 0.14 g dry mass mg^−1^ P), intermediate in MP plants (1.0 ± 0.10 g dry mass mg^−1^ P) and least in HP plants (0.14 ± 0.01 g dry mass mg^−1^ P with *p*_(treatment)_ ≤ 0.05).

**Table 1:** In planta P and P uptake characteristics of P deficient poplar

		Total P concentration [mg g^−1^]	Total P content [mg fraction^−1^]	^33^P recovery [% fraction^−1^]	P uptake [nmol fraction ^−1^]
HP	Leaf	6.80 ± 0.51^de^	8.55 ± 0.33^e^	6.79 ± 0.29^j^	43.4 ± 1.82^g^
	Stem	4.92 ± 0.55^d^	2.56 ± 0.20^d^	1.35 ± 0.15^e^	8.64 ± 0.96^f^
	Fine Root	11.27 ± 1.40^e^	4.79 ± 1.49^de^	5.79 ± 0.97^i^	37.01 ± 6.20^g^
	Coarse Root	6.98 ± 1.41^de^	1.92 ± 0.32^d^	1.04 ± 0.10^d^	6.65 ± 0.65^f^
MP	Leaf	1.25 ± 0.10^c^	0.19 ± 0.03^bc^	4.51 ± 0.38^h^	0.29 ± 0.02^d^
	Stem	1.11 ± 0.11^bc^	0.11 ± 0.02^abc^	7.22 ± 0.79^k^	0.46 ± 0.05^de^
	Fine Root	0.67 ± 0.12^abc^	0.08 ± 0.02^ab^	10.07 ± 0.69^l^	0.64 ± 0.04^e^
	Coarse Root	1.03 ± 0.15^bc^	0.23 ± 0.03^c^	3.73 ± 0.79^g^	0.24 ± 0.05^d^
LP	Leaf	0.69 ± 0.03^abc^	0.15 ± 0.03^bc^	0.08 ± 0.02^a^	0.059 × 10^−3^ ± 0.001 × 10^-3 a^
	Stem	0.62 ± 0.17^ab^	0.05 ± 0.02^a^	0.12 ± 0.03^b^	0.088 × 10^−3^ ± 0.003 × 10^-3 a^
	Fine Root	0.41 ± 0.10^a^	0.05 ± 0.01^a^	1.94 ± 0.47^f^	1.48 × 10^−3^ ± 0.35 × 10^-3 c^
	Coarse Root	0.49 ± 0.03^ab^	0.10 ± 0.01^abc^	0.41 ± 0.19^c^	0.32 × 10^−3^ ± 0.15 × 10^-3 b^
*p*-value	*p*_(treatment)_	<0.001	<0.001	<0.001	<0.001
*p*-value	*p*_(fraction)_	0.225	<0.001	<0.001	<0.001
*p*-value	*p*_(treatment×fraction)_	<0.01	<0.001	<0.001	<0.001
HP	whole plant	7.10 ± 0.58^C^	17.82 ± 1.38^C^	14.98 ± 1.36^B^	95.69 ± 8.71^C^
MP	whole plant	1.03 ± 0.11^B^	0.61 ± 0.05^B^	25.52 ± 1.84^C^	1.63 ± 0.12^B^
LP	whole plant	0.56 ± 0.04^A^	0.34 ± 0.06^A^	2.55 ± 0.64^A^	1.94 × 10^−3^ ± 0.49 × 10^-3 A^
*p*-value		<0.001	<0.001	<0.001	<0.001

Total P concentration and total P content per dry mass, ^33^P recovery and calculated uptake of P after 2 days in tissues of and whole poplar plants grown with one of three different P concentrations in the nutrient solution in sand (HP: 641 μM, MP: 6.4 μM, LP: 0.064 μM). Different letters indicate significant differences (*p* ≤ 0.05, Two-Way-ANOVA and Tukey’s honest significance test, mean ± SE, *n* = 4-5)

### P uptake is not energy limited

**Fig. 3:**
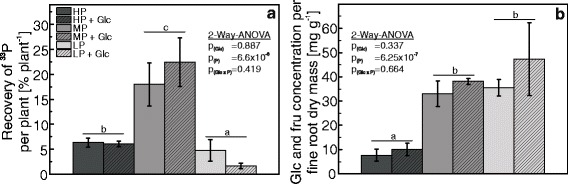
^33^P uptake and carbohydrate concentrations in P deficient poplar supplied with glucose in the nutrient solution. **a** The recovery of ^33^P in whole plants relative to the total amount of ^33^P added to the nutrient solution. **b** Soluble carbohydrate concentrations as the sum of fructose and glucose in roots of poplars. The plants were grown with three different phosphate concentrations in the nutrient solutions (HP: 641 μM, MP: 6.4 μM, LP: 0.064 μM) that were supplemented with 400 mM glucose for 2 h before adding 1 mL ^33^P labeled nutrient solution. Controls received no glucose. Different letters indicate significant differences between treatments (*p* ≤ 0.05, Two-Way-ANOVA and Tukey’s honest significance test, *n* = 5, mean ± SE)

Because LP plants showed lower relative P uptake than MP or HP plants, we tested whether P uptake was inhibited by energy depletion. For this purpose, the growth medium was supplemented with glucose before adding the ^33^P-labeled nutrient solution. There was no difference in P uptake between the glucose-fed and the non-fed control plants. As before, MP plants showed the highest, HP intermediate and LP plants the lowest P recovery (Fig. 3a), but the overall levels were lower because we used only 14 h exposure time instead of 48 h to avoid confounding effects of microbial growth. The soluble carbohydrate concentrations in fine roots were unaffected by glucose treatment (Fig. 3b). The concentration of soluble carbohydrates was about 4-fold higher in the fine roots of P-depleted poplars (MP, LP) than in HP plants (Fig. 3b).

### Low P concentrations in the nutrient solution limit P uptake

**Fig. 4:**
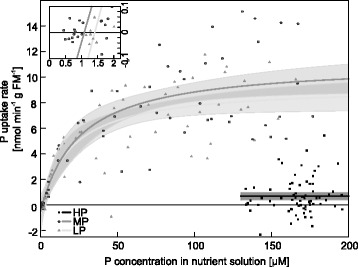
Model of kinetic parameters for P uptake of P deficient poplars at different P concentrations. P uptake rate per gram of fine root mass of plants grown with different P concentrations in the nutrient solution (black squares: HP, 641 μM; open circles: MP, 6.4 μM; grey triangles: LP, 0.064 μM) during exposure to different P concentrations. Data points from six different plants per treatment. Modeled curves (HP: black, MP: dark grey, LP: light grey) with parameters from Table 2 and 95 %-prediction interval in grey. The insert shows an enlargement of the curves at intermediate and low P concentrations around the concentration of C_min_

To investigate plant acclimation to decreasing P availabilities in the nutrient solution, P uptake kinetics of HP, MP, and LP plants were determined. The modeled Michaelis-Menten-curves for the P uptake of LP and MP plants showed a steep increase of the P uptake rate at low P concentrations in the nutrient solution and leveled off to different maximum uptake rates (Fig. 4, Table 2). The highest P uptake rate was found in MP plants and was similar to that of the LP plants (Table 2). The maximum P uptake rate was more than 13 times higher in LP plants than in the HP plants (Table 2, Fig. 4). The K_m_ was 25.9 (±9.9) μM in the MP and 19.9 (±8.1) μM in the LP plants. The minimum concentration C_min_, at which net uptake took place, was reached at 1.1 μM (Fig. 4, insert) and was similar for LP and MP poplars. HP plants exhibited very slow P uptake kinetics and therefore determination of K_m_ and C_min_ was not possible under our experimental conditions, where the maximum P concentration tested was 200 μM.

**Table 2:** Kinetic parameters for P uptake of P deficient poplar

	HP	MP	LP
v_max_ [nmol min^−1^ g^−1^]	0.689 (±0.164)^a^	11.140 (±1.065)^b^	9.311 (±0.908)^b^
K_m_ [μM]	n.d.	25.87 (±9.87)^ns^	19.85 (±8.11)^ns^
C_min_ [μM]	n.d.	1.08 (±0.91)^ns^	1.41 (±1.19)^ns^

Kinetic parameters (estimate ± standard error) for the uptake of P in plants grown with one of three different P concentrations in the nutrient solution in sand (HP: 641 μM, MP: 6.4 μM, LP: 0.064 μM). Model calculated with data from 6 different plants per treatment. Different letters in rows indicate significant differences at *p* ≤ 0.05. v_max_: maximum uptake rate, K_m_: Michaelis-Menten constant, C_min_: minimal concentration needed for uptake, n.d.: not determined

### Low P availabilities lead to differential regulation of P transporters

**Fig. 5:**
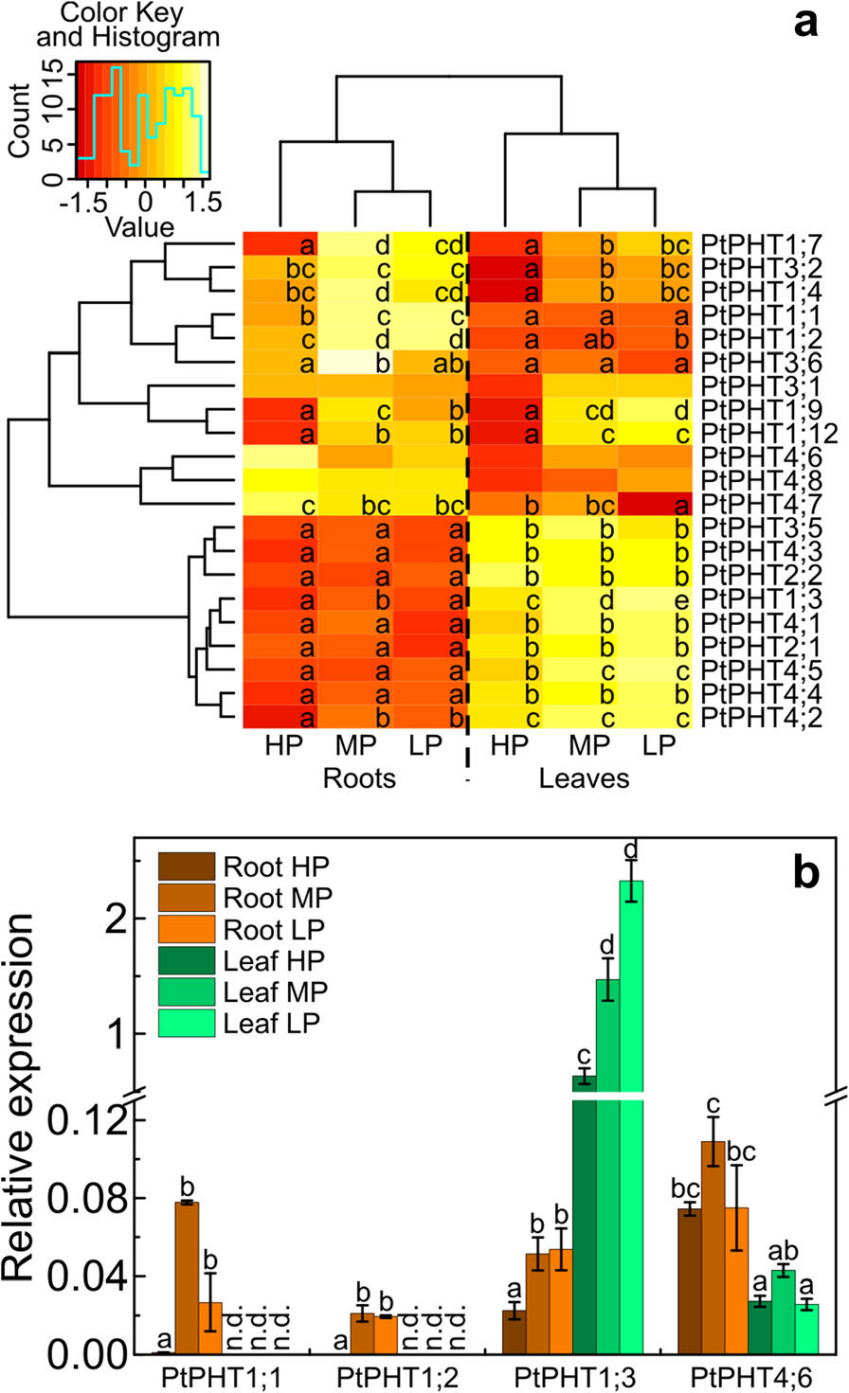
Expression of phosphate transporters in P deficient poplars. **a** Heatmap of the relative expression of putative phosphate transporter genes in the three uppermost leaves and fine roots of poplar plants grown with different P concentrations in the nutrient solution (HP: 641 μM, MP: 6.4 μM, LP: 0.064 μM). Red colors indicate lower and yellow to white colors higher values after z-transformation of the transcript abundances of each gene across all analyzed tissues and P conditions. **b** Relative expression of selected *PtPHT*s measured by qRT PCR (mean ± SE). Values were normalized to the expression of two reference genes (Actin, PPR-repeat) in the samples. Different letters indicate significant differences in expression of each gene (adj. *p* ≤ 0.05, ANOVA and Tukey’s honest significance test, *n* = 3)

As the pre-requisite to analyze the response of poplar P transporters from different families, we annotated all putative PHTs in the poplar genome based on homology searches using *Arabidopsis*, rice and maize (Additional file 1: Figure S2, Table S1). We found a total of 36 genes in poplar belonging to 4 different clades, of which 31 had probe sets on Affymetrix microarrays. Among those genes, 21 were expressed in roots and leaves (Fig. 5a, Additional file 1: Table S1, Table S3). We selected four genes for validation of their expression in different tissues and in response to different P supply (Fig. 5b) and found a strong correlation between qRT PCR and microarray data (Additional file 1: Figure S3).

The expression pattern of the 21 PHT genes clearly distinguished roots and leaves (Fig. 5a). *PtPHT1;1*, *PtPHT1;2*, *PtPHT1;4*, *PtPHT1;7*, *PtPHT1;9*, *PtPHT1;12*, *PtPHT3;1*, *PtPHT3;2*, *PtPHT3;6*, *PtPHT4;6*, *PtPHT4;7* and *PtPHT4;8* exhibited generally higher transcript levels in roots than in leaves (Fig. 5a). In leaves, the transcript abundances of *PtPHT1;3*, *PtPHT2;1, PtPHT2;2, PtPHT3;5*, *PtPHT4;1, PtPHT4;2, PtPHT4;3, PtPHT4;4* and *PtPHT4;5* were generally higher than in roots (Fig. 5a). It was notable that among the putative plasma membrane P transporters only *PtPHT1;3* was higher expressed in leaves than in roots and with almost 10-fold higher transcript abundance than in roots (Fig. 5b). The expression of *PtPHT1;1* and *PtPHT1;2* was root-specific because we could not detect any signal in leaves with specific primers in the qRT PCR analysis (Fig. 5b) and on microarrays the measured transcript abundances of these genes in leaves were classified with the expression probability “absent” (for the definition, see materials and methods). The putative chloroplastic P transporters (*PtPHT2;1*, *PtPHT2;2*) showed higher expression levels in leaves than in roots.

The transcript levels of most PHTs, which had been classified into the “root” group showed strong responsiveness to P starvation in both tissues, roots and leaves, whereas the PHT members classified into the “leaf” group did not respond to variation in P supply, with few exceptions (*PtPHT1;3*, *PtPHT4;2*, *PtPHT4;5*) (Fig. 5a). It is also important to note that the PHT members *PtPHT1;4, PtPHT1;7*, *PtPHT1;9,* and *PtPHT3;6* showed the highest transcript abundances in roots under MP and in leaves under LP conditions (Fig. 5a).

Our annotation of the PHT family members also uncovered P1BS (PHR1 binding sequence) elements, known to be involved in the P response [51] in the upstream region of the genes (Additional file 1: Table S1). P1BS elements were mainly present in PHT1 promotors (Additional file 1: Table S1). Among the P starvation responsive genes in family PHT1, *PtPHT1;1*, *PtPHT1;4*, *PtPHT1;7* and *PtPHT1;9* had a P1BS element in the 1 kb promotor region and in family PHT4 it was present in the promoter region of for *PtPHT4;5.* However, the P1BS element was not found in the 1 kb upstream region of other P starvation responsive genes of the PHT1, 3 and 4 families: *PtPHT1;2*, *PtPHT1;3, PtPHT1;12*, *PtPHT3;2*, *PtPHT3;6*, and *PtPHT4;2*, while it was present in *PtPHT3;1,* a gene, which showed no P starvation response under our experimental conditions.

## Discussion

### P uptake and allocation

Here, we characterized the uptake, allocation and PHT expression levels of *Populus* × *canescens* grown under different P availabilities. Similar as in *P. tremuloides* [25], we found that P availabilities below 60 μM resulted in strong reduction in height growth and biomass. These reductions were accompanied by a strong decline in tissue phosphorus concentrations, with the strongest decline in fine roots. The phosphorus concentrations found in leaves and fine roots of MP poplars in our study were similar to those found in conventional poplar plantations, as reported for example by Ge et al. [52]. Poplar plantations are often established on marginal sites and therefore information on the uptake characteristics and allocation in the trees is an essential pre-requisite for sustainable management [52]. Values indicating sufficient P supply of young *Populus* × *canescens* leaves range from 1.7 to 2.7 mg g^−1^ dry mass [53]. According to these values, the MP and LP poplars in our study suffered from strong and very strong deficiency, whereas the P concentrations in the HP poplars indicate luxurious P supply. Luxurious P concentrations cannot be used for growth and consequently, PUE of *P*. × *canescens* increased when the P availability declined as was also shown for other poplar species and crops [23, 54, 55]. It is further notable that the allocation pattern of newly taken up P was shifted towards leaves in the MP compared with LP or HP poplars. While usually the young leaves were the predominant aboveground sink, our imaging analyses showed that in MP plants also older leaves exhibited significant uptake of newly acquired P, resulting in an allocation shift. Nevertheless, no measurable newly acquired P was allocated to the oldest leaves. Belowground, the new P was mainly concentrated in those parts of root system that were close to the root-shoot junction, regardless of the P supply. This finding was surprising because under severe P limitation one may have expected preferentially allocation of P to growing tissues such as the fine root tips and the shoot apex. The observed P accumulation suggests that the root-shoot junction constitutes an obstacle to P below-aboveground translocation. Studies on heavy metal transport (cadmium, zinc) suggested that the root-shoot junction serves as a control barrier for translocation of those elements into the shoot probably due to changes in the anatomical structure of the xylem at transition from root to shoot xylem [56]. The molecular and cellular basis for the supposed control function is unknown and must be addressed in future research. Our data suggest that an improved allocation from roots to the shoot could perhaps contribute to enhance PUE. This suggestion is currently speculative, but worthwhile to be addressed in future studies.

Under P starvation, the poplars showed not only differences in allocation and an increased P use but also higher uptake efficiency than the well-supplied trees, as indicated by the increased recovery of ^33^P from the nutrient solution in MP compared with HP plants. While this finding is in agreement with crops and *Arabidopsis* [17–20, 57], severely P-starved poplars were not able to increase the relative P uptake compared with that of HP plants and even exhibited a significant decline in uptake efficiency. We considered whether energy limitation might have resulted in the reduction of P uptake efficiency. Because phosphorus occurs in the environment often in bound forms, P-starved poplars enhance the production and exudation of organic acids to increase the mobilization of external P [25]. Consequently, large amounts of photo-assimilates are lost when the plants attempt to counteract P deficiency. Furthermore, P-starved plants accumulate carbohydrates in chloroplasts because the export of the carbon skeletons into the cytosol is inhibited [58]. Nevertheless, for some plants an increased carbohydrate translocation to the roots and even an accumulation in roots was shown under P deprivation [58]. Also here, an accumulation of carbohydrates in roots of both MP and LP compared with HP plants was found and exposure to additional glucose could not stimulate P uptake under any of the tested P concentrations in the growth medium. It is known that glucose is taken up by plants from the medium via monosaccharide transporters [59] into the cytoplasm of root cells, i.e., the same compartment into which sugars from source tissues are unloaded [60]. Therefore, the availability of sugar taken up from the medium does not differ from plant-derived sugar and thus, we could exclude energy limitation as the reason for the decrease in ^33^P-recovery of severely P-stressed poplars.

P uptake is further governed by the kinetic properties of the uptake systems. We found a threshold concentration of about 1 μM in the medium required for net P uptake. One reason for such a threshold could be the efflux of newly acquired ^33^P from the roots back into the medium at very low external P concentrations because of the steep concentration gradient. Another reason could be that the ratio of the monovalent form of P (H_2_PO_4_^−^), which is taken up by plants [9], was shifted to the acid form at the membrane due to an excess of protons under very low external P concentrations [61] and then was not available for plant uptake.

Here, we showed that the K_m_ for P uptake of the root system of MP poplars did not decrease significantly when the phosphate concentration in the nutrient solution was further decreased. This finding suggests that the uptake systems operate at their limits under these conditions. Desai et al. [25] found a similar K_m_ value of about 30 μM for P accumulation in *P. tremuloides* across a range of low P concentrations. The apparent K_m_ values for P uptake of P-starved poplars are, however, higher than phosphate concentrations in soil solutions in many non-fertilized ecosystems, for instance 5 μM in forests beneath the organic layer [62–65]. These soil concentrations are too low for sufficient P nutrition and the up-regulation of uptake systems would only ensure net uptake that occurred above the threshold concentration of 1 μM P. Thus, neither decreases in K_m_ values nor increases in v_max_ by up-regulation of phosphate transporters would be sufficient to combat P deficiency in natural environments [66]. Under field conditions poplar roots are colonized by mycorrhizal fungi with different abilities for P acquisition [67–71]. Mycorrhizal roots usually show lower K_m_ values for P uptake [72, 73] and higher tissue P concentrations than non-mycorrhizal roots [74]. In poplar the reduction in K_m_ of P accumulation after colonization with the mycorrhizal fungus *Laccaria bicolor* was moderate (about 6 μM instead of 30 μM [25]). In *Pinus sylvestris*, mycorrhizal colonization decreased not only the K_m_ from 12.1 μM to 3.5 μM, but also the minimum threshold concentration for net P uptake of 2 μM to 0.2 μM [73]. Altogether, these finding underpin that without the help of microbes, P nutrition of poplars is critical in nature and may often fall below the threshold for net uptake.

### Regulation of the poplar PHT families in response to P starvation

P uptake and intracellular distribution is achieved by four P transporter families, with different number of genes in different plant species. Our *in silico* annotation of the PHTs of poplars showed that the PHT1 family was the largest with 12 members, followed by PHT4 with 9 members, while PHT3 and PHT2 contained 6 and 2 members respectively. These findings demonstrate that not only poplar PHT1 [24], but also the other families exhibit an expansion compared to *Arabidopsis* [3, 61]. The PHT2, PHT3 and PHT4 families contain organelle membrane located transporters, which assure together photosynthetic and respiration processes [14]. The presence of the putative P responsive element P1BS in the 1 kb upstream region was confirmed for PHT1 family members and was further demonstrated for some members of the PHT3 and PHT4 families, but was unrelated to P starvation responsiveness as reported previously for the poplar PHT1 [24].

Here, the putative chloroplastic P transporters *PtPHT2;1* and *PtPHT2;2* exhibited low transcript abundance in roots and high in leaves, but were not responsive to P starvation. Similar results have been reported for the only member *AtPHT2;1* in *Arabidopsis* [6] suggesting that the functional properties have been maintained. The results for the mitochondrial PHTs are more divergent because *AtPHT3;1* was unresponsive to P starvation, whereas its homologs in poplar (*PtPHT3;1* and *PtPHT3;2*) showed increased transcript levels (this study; [75]). Poplar *PtPHT3;5*, an ortholog to the mitochondrial *Arabidopsis AtPHT3;3* was unresponsive to P deprivation in both plant species (this study; [75]).

The PHT4 family showed an interesting clustering with *PtPHT4;6*, *PtPHT4;7* and *PtPHT4;8*, the orthologs of *AtPHT4;6*, *AtPHT4;2* and *AtPHT4;3*, respectively, exhibiting higher transcript levels in roots than in leaves, and higher transcript abundances in leaves than in roots for *PtPHT4;1*, *PtPHT4;2*, *PtPHT4;3*, *PtPHT4;4* and *PtPHT4;5*, which are the orthologs to *AtPHT4;1*, *AtPHT4;4* and *AtPHT4;5*. An expression pattern similar to that in poplar was reported for *Arabidopsis* with higher transcript levels in roots for *AtPHT4;6*, and *AtPHT4;2* and higher levels in leaves for *AtPHT4;1*, *AtPHT4;4* and *AtPHT4;5* [3]. The latter transporters were localized to chloroplasts, while AtPHT4;6 was localized in the Golgi membrane [3]. In poplar, the members of PHT4 were not or only little regulated by P starvation. Still, they have important function for intracellular trafficking of P. For example, deletion of the Golgi-membrane localized AtPHT4;6 leads to severe growth impairment and symptoms of P starvation, despite normal tissue P concentrations [76]. Altogether, similar tissue-specific expression pattern and P responsiveness of many organelle-related P transporters suggest that their functions are conserved in *Arabidopsis* and poplar.

The members of PHT1 family are localized to the plasma membrane and catalyze P uptake from the soil and long distance transport in plants [5, 61, 77]. Each of the nine *Arabidopsis* and all analyzed poplar PHT1 members show increased transcript abundance after exposure to low P supply (this study; [24, 75]), although some genotype-related exception were found [24]. While three of the nine *Arabidopsis* PHT1 genes (*AtPHT1;3*, *AtPHT1;5*, *AtPHT1;6*) exhibited higher expression in leaves than in roots [75], in poplar, only *PtPHT1;3* showed this pattern. *PtPHT1;3* was phylogenetically related to *AtPHT1;4* and *AtPHT1;7*, but showed a higher distance to these *Arabidopsis* genes than the poplar genes *PtPHT1;4*, *PtPHT1;5* and *PtPHT1;7*. This observation suggests that *PtPHT1;3* may have evolved more rapidly and already acquired additional functions compared to the *Arabidopsis* genes.

The expression patterns of poplar *PtPHT1;9* and *PtPHT1;12* were similar in roots and in leaves with activation in response to P deficiency. Their orthologs in *Arabidopsis* (*AtPHT1;9* and *AtPHT1;8* for *PtPHT1;9*) mediated high-affinity P uptake in roots [78] and *AtPHT1;5* (for *PtPHT1;12*) was located in the vascular system [79] suggesting important roles for uptake and long-distance transport under P starvation. The expression of these transporters was most strongly stimulated by P deficiency in *P. trichocarpa* [24]. In contrast to those results, we found the strongest responsiveness to P starvation for *PtPHT1;1*, *PtPHT1;2*, *PtPHT1;12* and *PtPHT1;7*, which underline genotype-related differences in the expression patterns. In *Arabidopsis*, *AtPHT1;1* and *AtPHT1;4*, orthologs to those poplar genes, are expressed in the epidermal layer of roots and mediate high-affinity root uptake [16, 79, 80]. The pre-dominant expression of those poplar transporters in roots and their strong root-specific up-regulation upon P limitation, suggests that these functions are conserved in *P.* × *canescens* and *Arabidopsis thaliana*.

## Conclusions

Here, we annotated the four PHT families in poplar and demonstrated tissue-specific expression patterns in response to P starvation. P transporters that were higher expressed in young leaves than in fine roots belonged to the organelle-related families and showed no response to P starvation, with the exception of *PtPHT1;3*. The transcript abundance of the latter transporter in leaves was high under high P supply, but further increased massively under P starvation suggesting an important role of this gene for foliar P supply in accordance with highest P allocation to the youngest leaves. Other members of the PHT1 family also showed strong up-regulation in response to P starvation, but were generally higher expressed in roots than in leaves underpinning their role in P uptake from the environment. The root-specific increment in *PtPHT1;1* and *PtPHT1;2* expression suggests a major role for these transporters in combating P deficiency in poplar.

In accordance with the enhanced expression of PHT members in roots, P uptake efficiency was enhanced at the intermediate P level, which represents a concentration that can occur in soil solution. Furthermore, P starved poplars showed the highest allocation of newly acquired P to fine roots, suggesting that these organs were preferentially supplied with P to maintain their physiological activities.

We found that the threshold for net P uptake was about 1 μM and the apparent K_m_ for the whole-root system about 26 μM. These findings indicate that under low P availabilities, which occur often in soil solutions, bare-rooted poplars are prone to suffer from P limitations. Mycorrhizal colonization of the roots can increase net P uptake [81], but which fungal species are the most useful to combat P deficiency is an open question, which needs to be addressed for sustainable poplar plantation management.

## Additional file

Additional file 1:
**Table S1.** In silico analyses of putative poplar phosphate transporters; **Table S2.** Primers used for qRT PCR of putative P transporter genes; **Table S3**. Transcript abundances of phosphate transporter genes; **Figure S1.** Biomass and performance of poplar grown with five different P concentrations; **Figure S2.** Neighbor-Joining tree of the amino acid sequences for inorganic phosphate transporters in poplar; **Figure S3.** Correlations of absolute microarray expression data (log_2_-value) and qRT PCR relative expression values (log_2_) for PtPHTs. (PDF 1255 kb)

## Abbreviations

HP: High phosphate
LP: Low phosphate
MP: Medium phosphate
P: Phosphorus
PHT: Phosphate transporters
P_i_: Inorganic phosphate
PUE: P use efficiency
qRT PCR: Quantitative Real Time PCR
