# Supplementary material for: Phosphate uptake kinetics and tissue-specific transporter expression profiles in poplar (Populus × canescens) at different phosphorus availabilities
Source: BMC Plant Biol. 2016 Sep 23;16:206. doi: 10.1186/s12870-016-0892-3 (PMC5035498; doi:10.1186/s12870-016-0892-3)
Supplement: Additional file 1: — Table S1. In silico analyses of putative poplar phosphate transporters; Table S2. Primers used for qRT PCR of putative P transporter genes; Table S3. Transcript abundances of phosphate transporter genes; Figure S1. Biomass and performance of poplar grown with five different P concentrations; Figure S2. Neighbor-Joining tree of the amino acid sequences for inorganic phosphate transporters in poplar; Figure S3. Correlations of absolute microarray expression data (log2-value) and qRT PCR relative expression values (log2) for PtPHTs. (PDF 1255 kb) [file 12870_2016_892_MOESM1_ESM.pdf]

Table S1: In silico analyses of putative poplar phosphate transporters.

GeneID: Poplar gene IDs in genome annotation version v3, PLACE P1BS-Localization: location of P1BS-motif(s) in 1 kb upstream region of the gene, Affy IDs: probe names on annotated for the gene by Tsai et al. [1], Ath protein homolog: closest Arabidopsis protein homolog after Figure S1 and its identity (%) with the poplar protein, n.d.: expression not detected on microarray, NA: gene is not on array, tr.: truncated; uncl: unclustered.

**GeneID**	**Gene name or Family**	**PLACE** P1BS-Localization	**Affy IDs**	**Comments**	**Ath protein homolog (% identity)**
Potri.010G072000	*PtPHT1;1*	54	PtpAffx.215047.1.S1_s_at<br>PtpAffx.208712.1.S1_at<br>PtpAffx.219484.1.S1_s_at		AtPHT1;5 (77.5)
Potri.010G071700	*PtPHT1;2*		PtpAffx.219485.1.S1_s_at		AtPHT1;5 (78.7)
Potri.010G071500	*PtPHT1;3*		PtpAffx.54440.1.A1_at		AtPHT1;4 (82.5),<br>AtPHT1;7 (82.0)
Potri.005G223500	*PtPHT1;4*	404	PtpAffx.141538.1.S1_s_at<br>Ptp.5181.2.S1_at<br>PtpAffx.69535.1.A1_s_at		AtPHT1;4 (83.0),<br>AtPHT1;7 (84.7)
Potri.002G038900	*PtPHT1;5*	169		NA	AtPHT1;4 (84.5),<br>AtPHT1;7 (83.6)
Potri.005G175500	*PtPHT1;6*		PtpAffx.223352.1.S1_s_at	n.d.	AtPHT1;6 (64.5),<br>At4G08895 (53.3)
Potri.005G223600	*PtPHT1;7*	911	PtpAffx.205756.1.S1_x_at<br>PtpAffx.205756.1.S1_at<br>PtpAffx.69535.2.A1_s_at	 n.d.	AtPHT1;4 (85.9),<br>AtPHT1;7 (86.9)
Potri.019G061900	*PtPHT1;8*		PtpAffx.215592.1.S1_at<br>PtpAffx.223434.1.S1_at<br>PtpAffx.223434.1.S1_s_at	n.d.<br>n.d.<br>n.d.	-
Potri.002G005500	*PtPHT1;9*	334	PtpAffx.62481.1.A1_s_at<br>PtpAffx.201523.1.S1_at	 n.d.	AtPHT1;8 (60.4),<br>AtPHT1;9 (62.9)
Potri.015G022800	*PtPHT1;10*	489, 581, 826	PtpAffx.218285.1.S1_at	n.d.	-
Potri.005G256100	*PtPHT1;11*	514		NA	AtPHT1;8 (61.0),<br>AtPHT1;9 (64.2)
Potri.001G318500	*PtPHT1;12*		PtpAffx.201089.1.S1_at		AtPHT1;5 (78.9)
Potri.005G175700	Fam. 1		PtpAffx.205527.1.S1_at	n.d.	AtPHT1;6 (66.5),<br>At4G08895 (53.3)
Potri.005G223700	Fam. 1		PtpAffx.205757.1.S1_at	n.d., tr.	-
Potri.010G071600	Fam. 1			NA	AtPHT1;5 (78.9)
Potri.013G089800	Fam. 1	449, 472		NA, tr.	-
Potri.008G186600	*PtPHT2;1*		PtpAffx.208289.1.S1_at		AtPHT2;1 (81.7)
Potri.010G046300	*PtPHT2;2*		PtpAffx.208671.1.S1_at		AtPHT2;1 (73.3)

Table S1: In silico analyses of putative poplar phosphate transporters (continued)

**GeneID**	**Gene name or Family**	**PLACE** P1BS-Local-ization	**Affy IDs**	**Com-ments**	**Ath protein homolog (% identity)**
Potri.017G060800	*PtPHT3;1*	305	PtpAffx.223426.1.S1_at		AtPHT3;1 (88.3)
			PtpAffx.51641.2.S1_at	n.d.	
Potri.001G322300	*PtPHT3;2*		PtpAffx.7845.1.A1_s_at		AtPHT3;1 (88.8)
			Ptp.2106.1.A1_s_at		
			PtpAffx.51641.1.S1_s_at		
			PtpAffx.201077.1.S1_at		
Potri.012G105100	*PtPHT3;3*			NA	-
Potri.015G104400	*PtPHT3;4*	277	Ptp.6141.1.S1_at	n.d.	-
Potri.004G207200	*PtPHT3;5*		PtpAffx.172.1.S1_at		AtPHT3;3 (75.2)
			PtpAffx.222497.1.S1_s_at		
Potri.005G098800	*PtPHT3;6*		PtpAffx.205286.1.S1_at		AtPHT3;3 (72.7)
			PtpAffx.13003.1.S1_at		
Potri.006G062300	*PtPHT4;1*		PtpAffx.216795.1.S1_at		AtPHT4;5 (71.7)
			Ptp.850.1.S1_at	n.d.	
Potri.018G121600	*PtPHT4;2*		PtpAffx.214529.1.S1_at		AtPHT4;5 (67.0)
			PtpAffx.214529.1.S1_x_at		
			PtpAffx.157223.1.S1_at		
			Ptp.6820.1.S1_at		
Potri.001G249800	*PtPHT4;3*		Ptp.7586.1.S1_at		AtPHT4;1 (75.6)
Potri.009G043800	*PtPHT4;4*		PtpAffx.95515.1.S1_at		AtPHT4;1 (75.4)
Potri.014G085700	*PtPHT4;5*	958	PtpAffx.59699.2.A1_a_at		AtPHT4;4 (73.8)
Potri.009G168200	*PtPHT4;6*		PtpAffx.147587.1.A1_at	n.d.	AtPHT4;6 (78.7)
			PtpAffx.204370.1.S1_at		
Potri.016G111000	*PtPHT4;7*		Ptp.5773.1.S1_at		AtPHT4;2 (73.8)
Potri.001G248200	*PtPHT4;8*		PtpAffx.107855.1.A1_a_at		AtPHT4;3 (77.0)
			PtpAffx.112006.1.S1_s_at	n.d.	
			PtpAffx.107855.1.A1_at		
			PtpAffx.200753.1.S1_at		
			PtpAffx.112006.1.S1_at	n.d.	
Potri.009G042000	*PtPHT4;9*		PtpAffx.204925.1.S1_at	n.d.	AtPHT4;3 (64.3)
Potri.006G109800	Fam. 4	434	PtpAffx.206360.1.S1_at	n.d., tr.	-
Potri.004G061400	uncl.		PtpAffx.203816.1.S1_at	n.d.	-
Potri.013G095600	uncl.		PtpAffx.211244.1.S1_at	n.d., tr.	-

Table S2: **Primers used for qRT PCR of putative P transporter genes**.

Genes are indicated by their Potri.IDs, Affy IDs for probe sets on microarray, indication of special design, and reference. When published primers were not available, they were designed with OligoExplorer 1.5 and checked with OligoAnalyzer 1.5 (both Gene Link, Hawthorne, NY, USA). Primers were adapted to *P*. × *canescens* (sequences from AspenDB) when the nucleotide sequence differed from *P*. *trichocarpa*.

Potri.ID	Affy IDs	Primer name	Primer sequence (5'-3')	Design	Reference
Potri.010G072000 (PtPHT1;1)	PtpAffx.215047.1.S1_s_at<br>PtpAffx.208712.1.S1_at<br>PtpAffx.219484.1.S1_s_at	PtPH1f<br>PtPH1r	GCGATTCACGAGGTTTTTCA<br>GGCGAAGAAAAAGGTCAACG		Loth-Pereda et al. [2]
Potri.010G071700 (PtPHT1;2)	PtpAffx.219485.1.S1_s_at	PcPHT1;2_fw<br>PcPHT1;2_rev2	CACAGACCGAACGAAGACTG<br>ATCACACTGAAGCCATCCTAGG		(after) Loth-Pereda et al. [2]
Potri.010G071500 (PtPHT1;3)	PtpAffx.54440.1.A1_at	PcPHT1;3_fw<br>PcPHT1;3_rev	CGACAACCGAATTGGCTTCG<br>CAAGTGGACCTCAGTCTCG	intron spanning	after Loth-Pereda et al. [2]
Potri.009G168200 (PtPHT4;6)	PtpAffx.147587.1.A1_at<br>PtpAffx.204370.1.S1_at	PcPHT4;6_fw<br>PcPHT4;6_rev	CGCTCGCTGCCAATCTTAC<br>GTCTAGGGATGTTCCACCC	intron spanning	
Potri.001G047200 (PPR-repeat as reference gene)	PtpAffx.200189.1.S1_at	Pc001G047200_fw<br>Pc001G047200_rev	GGCTGAGGAATGTCGAATGG<br>AGAACGCAACATCATGGAAACC	Exon-Exon junction	
Potri.001G309500 (Actin as reference gene)	Ptp.6486.1.S1_s_at<br>PtpAffx.1258.1.S1_s_at<br>PtpAffx.1258.4.S1_s_at	Aktin9_fw<br>Aktin9_rev	TGGTGGTTCCACTATGTTCC<br>TGGAAATCCACATCTGCTGG	Exon-Exon junction	Janz et al. [3]

Table S3: Transcript abundances of phosphate transporter genes

Raw expression values for the PHT genes on microarrays (means of n = 3) used for z-normalization to construct the heatmap shown in Figure 5a. Poplar plants were grown with nutrient solutions differing in phosphate concentration (641 µM P (HP), 6.4 µM P (MP), 0.064 µM P (LP)).

	Fine Roots			Uppermost Leaves		
	HP	MP	LP	HP	MP	LP
PtPHT1;1	44.6	3699.2	2721.1	6.2	6.7	7.2
PtPHT1;2	250.2	6658.6	6268.9	4.7	7.3	9.6
PtPHT1;3	18.0	35.5	22.5	386.1	818.6	1251.1
PtPHT1;4	196.7	423.4	287.6	70.7	179.9	199.1
PtPHT1;7	14.3	195.4	129.4	11.6	43.7	79.1
PtPHT1;9	20.6	169.2	84.2	14.1	195.4	304.6
PtPHT1;12	6.8	205.5	211.4	5.7	323.1	451.6
PtPHT2;1	7.3	7.0	5.8	15.5	18.0	19.0
PtPHT2;2	6.3	5.3	7.3	162.9	103.2	96.7
PtPHT3;1	101.2	99.8	98.3	78.8	106.2	103.3
PtPHT3;2	348.7	457.0	445.7	207.4	308.5	327.6
PtPHT3;5	6.7	7.7	6.4	18.4	21.6	16.6
PtPHT3;6	22.5	32.7	23.3	18.6	19.3	17.5
PtPHT4;1	54.4	63.6	48.7	112.1	146.1	156.3
PtPHT4;2	15.7	22.2	20.1	39.0	47.6	46.1
PtPHT4;3	4.5	7.1	5.4	33.1	34.0	34.1
PtPHT4;4	15.7	30.0	33.5	156.0	230.2	268.2
PtPHT4;5	21.6	18.5	25.1	215.4	616.5	728.6
PtPHT4;6	57.0	41.2	46.1	31.8	42.5	38.6
PtPHT4;7	134.4	118.8	119.2	90.3	101.8	63.5
PtPHT4;8	35.7	34.5	34.0	26.2	27.5	30.4

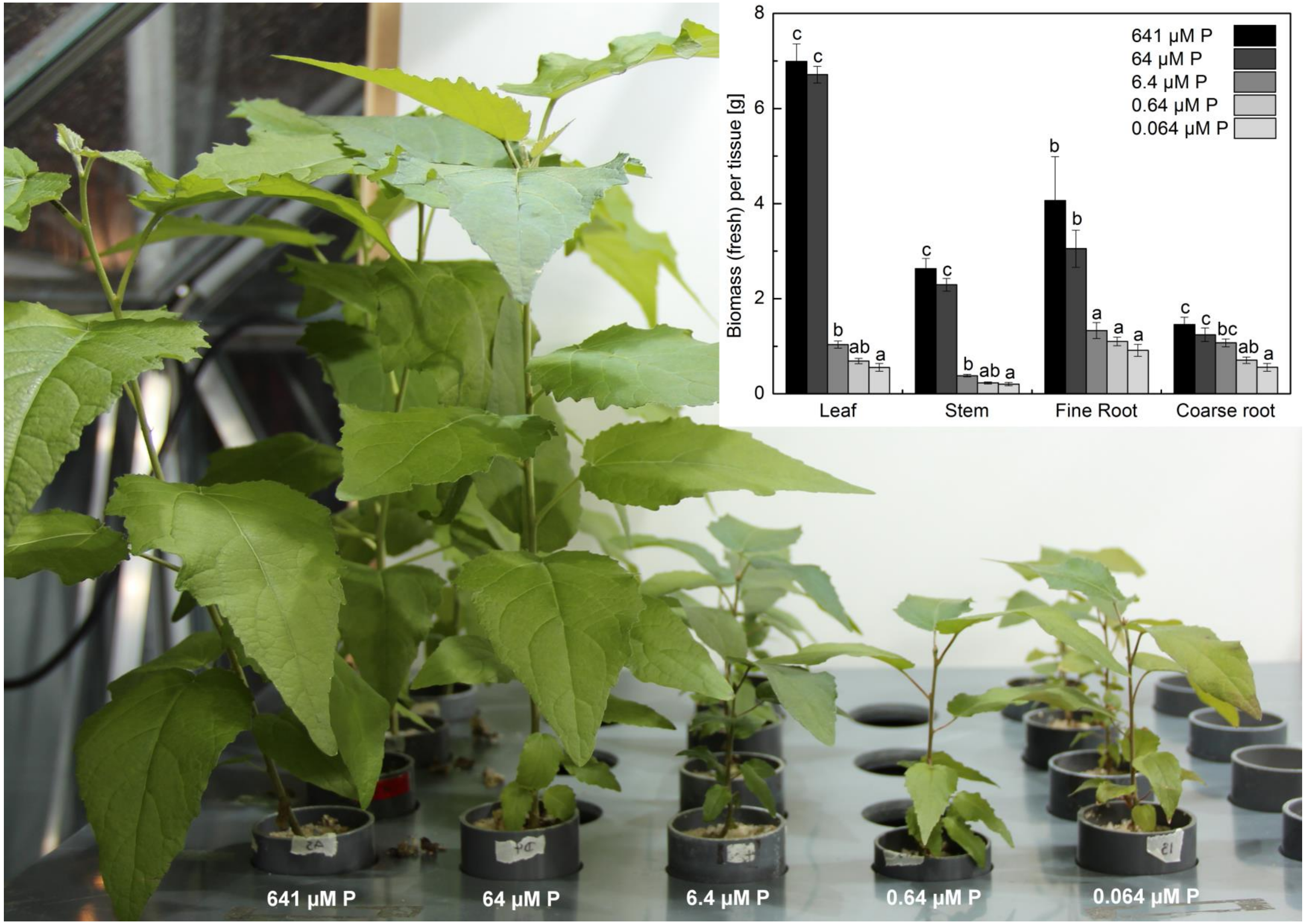

Figure S1: Biomass and performance of poplar grown with five different P concentrations.

Populus × *canescens* plants were grown for about 2 months in nutrient solutions differing in phosphate concentrations (641 µM P (HP), 64 µM P, 6.4 µM P (MP), 0.64 µM P, 0.064 µM P (LP)). Biomass data are means for n=11-12 (±SE).

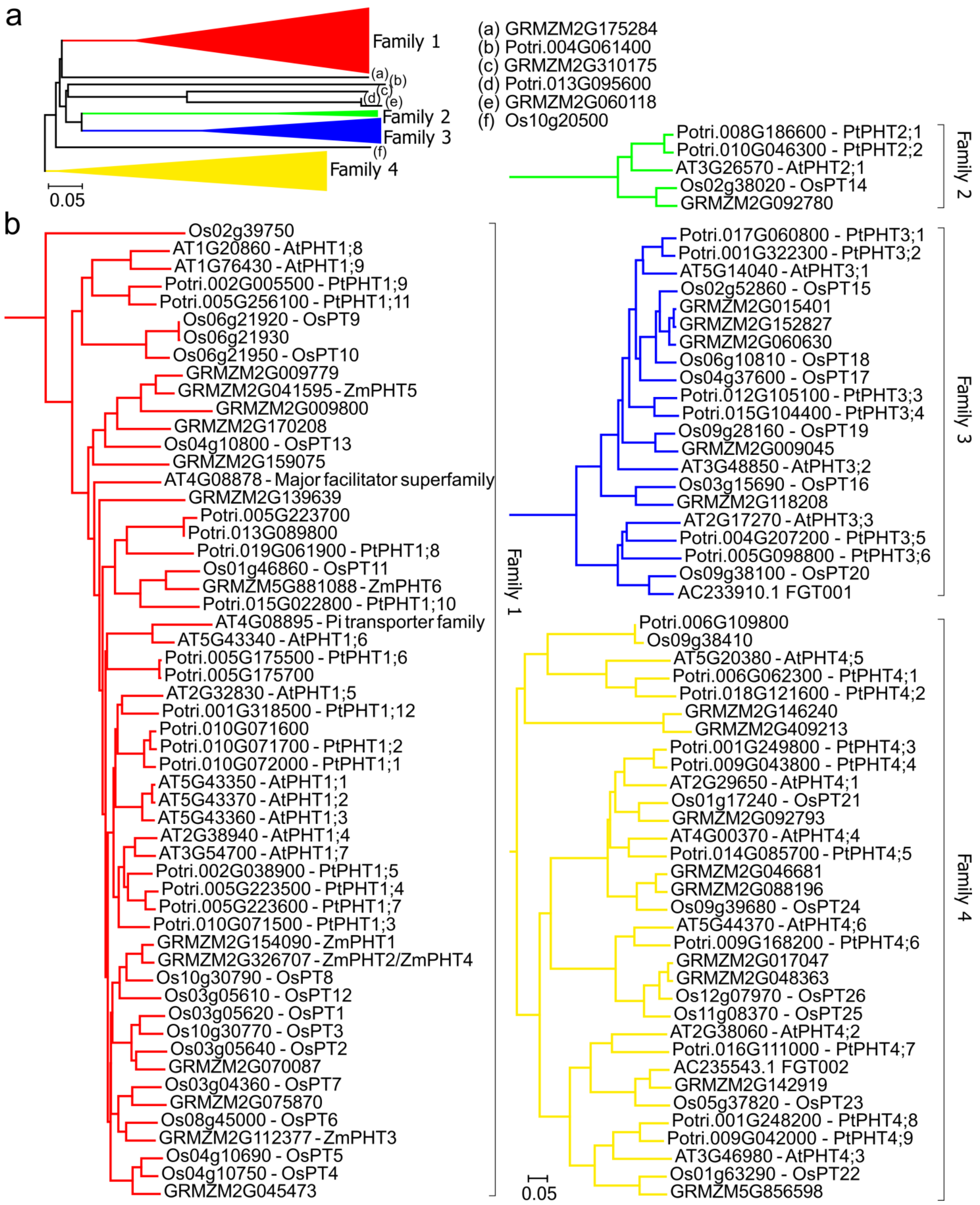

Figure S2: Neighbor-Joining tree of the amino acid sequences for inorganic phosphate transporters in poplar

Poplar (Potri), *Arabidopsis* (AT), rice (Os) and maize (GRMZM and AC) sequences were used; A: unrooted compressed phylogenetic tree; B: expanded view of the four families, names according to Fan et al. [4], Liu et al. [5] and Phytozome, names for newly annotated poplar genes according to tree structure.

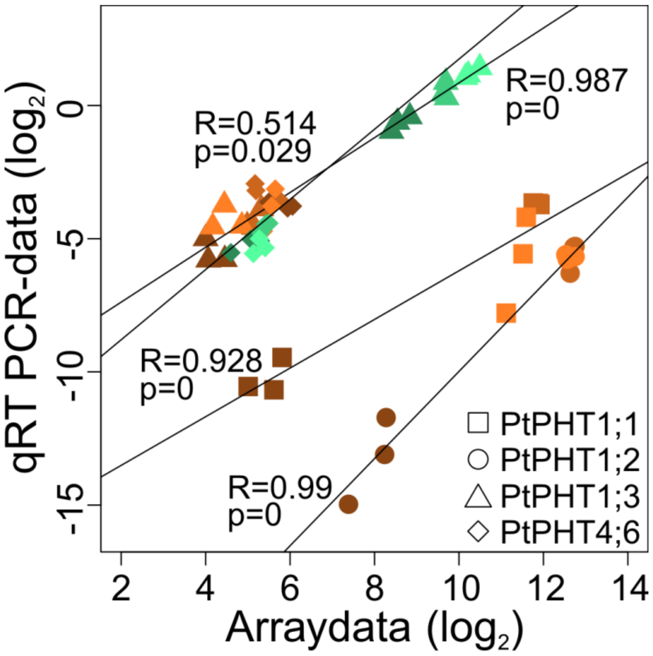

Figure S3: **Correlations of absolute microarray expression data (log_2-value) and qRT PCR relative expression values (log_2) for PtPHTs**

P-value and R-value of correlation (R function cor.test) are given, red: root samples, green: leaf samples, dark: HP, middle: MP, light: LP; expression of *PtPHT1;1* and *PtPHT1;2* could not be detected by qRT PCR in leaves. Absolute expression values are given for the microarray data and relative expression values to two reference genes (Actin, PPR-repeat) for qRT PCR data.

References

1. Tsai C-J, Ranjan P, DiFazio S, Tuskan G, Johnson V. Poplar genome microarrays. In: Joshi C, DiFazio S, Kole C, editors. Genet. Genomics Breed. Poplars. Enfield, NH: Science Publishers; 2011. p. 112–27.

2. Loth-Pereda V, Orsini E, Courty P-E, Lota F, Kohler A, Diss L, et al. Structure and expression profile of the phosphate Pht1 transporter gene family in mycorrhizal *Populus trichocarpa*. Plant Physiol. 2011;156:2141–2154.

3. Janz D, Lautner S, Wildhagen H, Behnke K, Schnitzler J-P, Rennenberg H, et al. Salt stress induces the formation of a novel type of “pressure wood” in two *Populus* species. New Phytol. 2012;194:129–41.

4. Fan C, Wang X, Hu R, Wang Y, Xiao C, Jiang Y, et al. The pattern of Phosphate transporter 1 genes evolutionary divergence in *Glycine max* L. BMC Plant Biol. 2013;13:48.

5. Liu F, Chang X-J, Ye Y, Xie W-B, Wu P, Lian X-M. Comprehensive sequence and whole-life-cycle expression profile analysis of the Phosphate transporter gene family in rice. Mol. Plant. 2011;4:1105–22.
